# Effects of an educational intervention on frailty status, physical function, physical activity, sleep patterns, and nutritional status of older adults with frailty or pre-frailty: the FRAGSALUD study

**DOI:** 10.3389/fpubh.2023.1267666

**Published:** 2023-11-30

**Authors:** Cristina Casals, Laura Ávila-Cabeza-de-Vaca, Andrea González-Mariscal, Alberto Marín-Galindo, Manuel Costilla, Jesus G. Ponce-Gonzalez, María Ángeles Vázquez-Sánchez, Juan Corral-Pérez

**Affiliations:** ^1^ExPhy Research Group, Department of Physical Education, Instituto de Investigación e Innovación Biomédica de Cádiz (INiBICA), Universidad de Cádiz, Cádiz, Spain; ^2^Department of Nursing, PASOS Research Group, Faculty of Health Sciences, UMA REDIAS Network of Law and Artificial Intelligence Applied to Health and Biotechnology, University of Malaga, Málaga, Spain

**Keywords:** functional capacity, educational program, community-dwelling, frail, accelerometry

## Abstract

**Introduction:**

The prevalence of frailty is increasing worldwide, emphasizing the importance of prioritizing healthy ageing. To address this, cost-effective and minimally supervised interventions are being sought. This study aimed to assess the impact of an educational program on frailty status, physical function, physical activity, sleep patterns, and nutritional status in community-dwelling older adults with at least 1 Fried’s frailty criteria.

**Methods:**

A 6-month multicentre randomized controlled trial was conducted from March 2022 to February 2023 in 14 health centres located in Cadiz and Malaga, Spain. The educational intervention consisted of 4 group sessions and 6 follow-up phone calls spread over 6 months. The program focused on educating participants about frailty and its impact on health, providing guidelines for physical activity, healthy dietary habits, cognitive training, psychological well-being and social activities. A total of 163 participants, divided into control (*n* = 80) and educational groups (*n* = 83) were assessed before and after the intervention.

**Results:**

The results showed a significant group-time interaction in the physical function evaluated with a large effect on Short Physical Performance Battery score (η2p = 0.179, −0.1 [−1.2–1.0] points for control group vs. 1.0 [0.0–3.0] points for educational group, *p* < 0.001), and an effect on the 4-meter gait test ((η2p = 0.122, 0.5 [0.1–0.0] s for control group vs. –0.4 [−0.5– −0.3] s for educational group, *p* < 0.001), and the 5-repetition sit-to-stand test (η2p = 0.136, 1.0 [0.0–1.2] s for control group vs. −4.3 [−7.0– −2.3] for educational group, *p* < 0.001). Additionally, the use of accelerometers to assess physical activity, inactivity, and sleep patterns revealed a significant small effect in the number of awakenings at night ((η2p = 0.040, 1.1 [−0.5–3.4] awakenings for control group vs. 0.0 [−2.2–0.0] awakenings for educational group, *p* = 0.009). The findings also highlighted a significant medium effect regarding malnutrition risk, which was assessed using the Mini-Nutritional Assessment score (η2p = 0.088, −0.7 [−2.3–1.5] points for control group vs. 1.5 [−0.5–3.0] points for educational group, *p* < 0.001).

**Discussion:**

Thus, the 6-month educational program effectively improved physical function, sleep patterns, and nutritional status compared to usual healthcare attendance in community-dwelling older adults with frailty or pre-frailty. These findings underscore the potential of minimally supervised interventions in promoting a healthy lifestyle in this vulnerable population.

## 1 Introduction

Frailty is characterized by the depletion of biological reserves, impaired physiological mechanisms, and susceptibility to a variety of adverse outcomes and it is closely related to ageing (1). Based on physical frailty, the prevalence of frailty and pre-frailty is 12 and 46% respectively, and according to the deficit accumulation model is 24% and 49%, respectively (2). Research has shown that older adults with frailty have a significantly higher risk of premature mortality compared to those without frailty, with individuals classified as pre-frail also facing an increased risk (3).

Frailty phenotype states and components exhibit dynamic longitudinal transitions (4). While frail is an important concern in the health care system, it is also possible to reduce adverse transitions, with frail individuals having an 18% probability of reverting to a pre-frail status (4). Thus, promoting healthy ageing should be a public health priority (5). In this regard, frailty preventive and risk factors include physical function, physical activity, sleep patterns, and nutrition (5–8), which are potentially modifiable through lifestyle interventions in community-dwelling older adults (9).

The progressive decline in physical function and the subsequent loss of personal self-sufficiency are classical concerns in actual geriatric medicine, closely associated with frailty. In recent years, there has been growing attention to the levels of physical activity and inactivity among older adults, which are often alarmingly low and have adverse health consequences (10, 11). Sleep patterns have also emerged as potential risk factors for frailty, as poor self-reported sleep quality and daytime drowsiness have been linked to a higher risk of frailty (12, 13). Additionally, nutrition plays a pivotal role in frailty maintenance. Malnourishment not only increases the risk of frailty but also elevates the chances of premature death and morbidity (13).

Previous studies have provided support for the effectiveness of exercise and nutritional interventions in reducing physical frailty (5, 7). However, social isolation (14) and cognitive training (15, 16) play also relevant roles in developing frailty. Therefore, there is a growing interest in exploring more comprehensive interventions that encompass all aspects, including physical, cognitive, and psychosocial factors, to effectively address and reduce frailty. These interventions should be designed to be implemented without excessive professional supervision promoting healthy ageing and aiming to search for the minimal intervention possible.

Therefore, the present study aimed to assess the effects of an educational program on frailty status, physical function, physical activity, sleep patterns, and nutritional status in community-dwelling older adults with frailty or pre-frailty. This educational program included guidelines for physical activity, nutrition, and cognitive training, as well as promoting psychological and social well-being over 6 months with 4 sessions and 6 phone calls, making it readily adaptable to nursing care routines.

## 2 Materials and methods

### 2.1 Design

A 6-month, multicentre, randomized controlled trial was conducted from March 2022 to February 2023 in 14 health centres located in the provinces of Cadiz and Malaga, Spain. All outcome measures were assessed before and after the 6-month intervention period. This trial was registered at ClinicalTrials.gov (Identifier: NCT05610605). The study was conducted following the principles of Good Clinical Practice Guidelines and the Declaration of Helsinki. Ethical approval was obtained from the Ethics Committee of the Provincial Research of Malaga (Approval code: FRAGSALUD). All participants were informed of the aims and characteristics of the study during a personal interview. After all participants gave written informed consent before enrolling in the study, Fried’s criteria were assessed, and if a participant met at least one criterion, all assessments were then conducted. The allocation of patients to the various study groups and the initial technique to be employed (either aspiration alone or an investigational technique) were randomized using the computer program Randomization^1^ in a block format with 80 participants per group.

### 2.2 Participants

The participants in this study consisted of community-dwelling individuals residing in both provinces. To qualify for participation, inclusion criteria were: being aged 65 years or older, and exhibiting at least one component of Fried’s frailty phenotype (17).

Exclusion criteria were individuals who were institutionalized, displayed symptoms of dementia during their clinical screening, or those who were categorized as robust (meaning they did not exhibit any component of Fried’s frailty phenotype).

**Figure 1:**
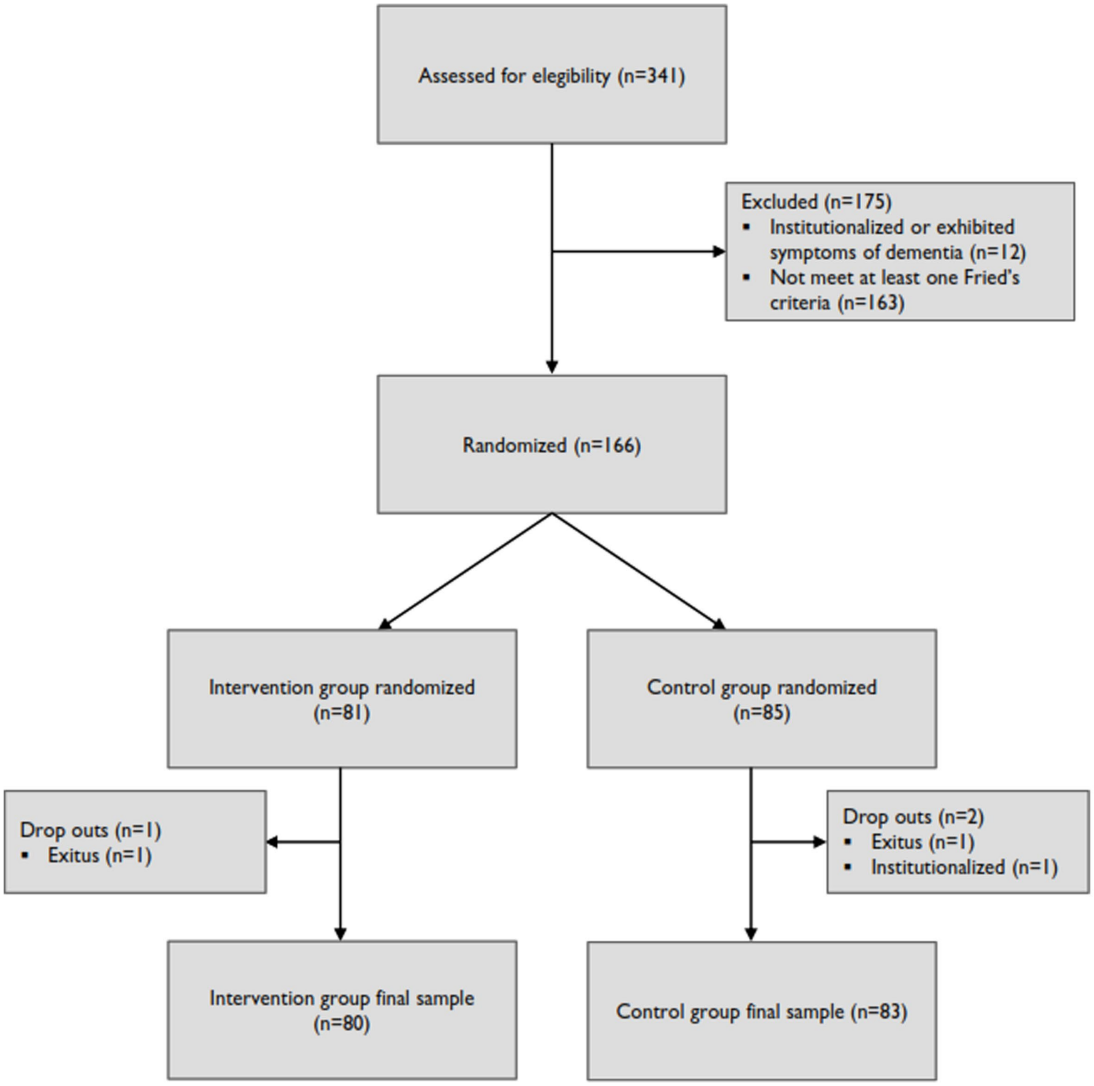
Flowchart of the FRAGSALUD project.

Out of the 166 participants initially included in the study, only 163 completed the post-intervention evaluation, with 2 participants from the control group and 1 from the educational group being lost to follow-up (Figure 1). The final analysis consisted of 80 participants in the educational group and 83 in the control group (Table 1).

**Table 1:** Baseline sociodemographic and medical characteristics of the frail/pre-frail older adults.

	Total (*n* = 163)	Control group (*n* = 83)	Educational group (*n* = 80)	*p*
Sex, *n* (%)				
Men	54 (33.1)	37 (44.6)	17 (21.3)	**0.001**
Women	109 (66.9)	46 (55.4)	63 (78.8)	
Age (years, median [IQR])	73.9 [69.4–78.9]	74.9 [70.8–80.7]	73.1 [68.5–76.5]	**0.019**
Height (cm, mean ± SD)	1.59 ± 0.09	1.62 ± 0.09	1.56 ± 0.09	**0.001**
Body mass	74.35 ± 14.90	75.96 ± 15.77	72.90 ± 14.41	0.16
Schoolarity, *n* (%)				
Less than primary school	25 (15.3)	12 (14.5)	13 (16.2)	**0.013**
Primary school	77 (47.2)	49 (59.0)	28 (35.0)	
Secondary school	39 (24.0)	15 (18.1)	24 (30.0)	
University and above	22 (13.5)	7 (8.4)	15 (18.8)	
Housing, *n* (%)				
Not alone	99 (62.7)	54 (68.4)	45 (57.0)	0.124
Alone	59 (37.3)	25 (42.4)	34 (43.0)	
Need for walking assistance, *n* (%)				
No	125 (80.1)	57 (74.0)	68 (86.1)	0.059
Yes	31 (19.9)	20 (26.0)	11 (12.8)	
Number of daily medication (number, median [IQR])	5.0 [3.0–8.0]	5.0 [3.3–8.3]	4.0 [3.0–7.0]	0.076
Smoking, *n* (%)				
Smoker	19 (11.7)	8 (9.6)	11 (13.8)	0.492
Non-smoker	144 (88.3)	75 (90.4)	69 (86.2)	
Alcohol drinks per week, *n* (%)				
None	120 (73.6)	67 (80.7)	53 (66.3)	0.111
Between 1 and 7	32 (19.6)	12 (14.5)	20 (25.0)	
More than 7	11 (6.8)	4 (4.8)	7 (8.8)	

IQR, interquartile range [percentile 25 – percentile 75]; SD, Standard Deviation. Significant differences are indicated in bold.

### 2.3 Intervention

A multidisciplinary team comprising nurses, nutritionists, and physical educators conducted the educational intervention. The educational group received a 6-month health education program consisting of 4 group sessions and 6 follow-up calls, while the control group maintained their usual healthcare attendance.

The 4 group sessions were conducted during the first month after the pre-test, with one session per week lasting approximately 45–60 min each. The educational sessions focused on the following topics: (1) frailty description and its impact on health, (2) guidelines for physical activity, (3) promoting healthy dietary habits, and (4) cognitive training and psychological and social well-being. Participants were encouraged to engage in debates and share their doubts and experiences. Each group session was limited to a maximum of 15 participants. If a participant was unable to attend a specific group session, another date with another group was scheduled to ensure a 100% attendance rate.

The aim of session 1 was to raise awareness about frailty, its implications on health, and how it progresses with ageing. During this session, emphasis was placed on the varying degrees of frailty, introducing and clarifying the concept of pre-frailty. Additionally, the session briefly addressed risk and preventive factors related to frailty, which would be further detailed in subsequent sessions. This session was imparted by a registered nurse.

Session 2 encompassed exercise recommendations for frail and pre-frail older adults, with a special emphasis on strength training in addition to the importance of aerobic exercise. The session highlighted the benefits of physical activity on health and quality of life to prevent the progression of frailty. Simple strength exercises that could be performed at home were demonstrated to improve muscle strength (e.g., squat to a chair or calf raises), along with guidance on exercises that should be avoided due to the risk of injury. Participants were provided with printed examples of exercises they could perform daily at home. Additionally, participants were encouraged to engage in physical and sports activities for older adults, either organized by private companies or the local municipality, while discouraging prolonged periods of sedentary behaviour. This session was performed by a sports scientist specialising in healthy physical exercise.

The main objective of session 3 was to promote dietary habits that would help prevent caloric-protein malnutrition, dehydration, and an imbalanced diet among the participants. Therefore, the Mediterranean diet was advocated, with special emphasis on certain aspects relevant to frail older adults, such as the daily consumption of a source of animal protein. Participants were encouraged to incorporate probiotics (e.g., yoghurt) and prebiotics (e.g., some vegetables) into their diets, with an explanation of the intestinal microbiota and its relationship to health. Printed examples were also provided for reference. Moreover, when frailty coexisted with another condition in which diet has a significant impact, such as diabetes, concepts like the glycaemic index were also covered, illustrating which foods or culinary techniques can increase it. This session was performed by a registered nutritionist.

Session 4 focused on the importance of cognitive training, psychological well-being, and social engagement. Participants performed some cognitive exercises and they were encouraged to incorporate them into their routines at least twice a week. These exercises included activities such as crosswords, word searches, Sudoku, personal diary writing, knitting or crocheting, painting, etc. Furthermore, they were motivated to engage in activities that pleased them, to recognize positive qualities within themselves, and to prioritize their mental well-being. Additionally, they were advised to participate in daily social activities to foster social engagement. This session was performed by a registered nurse.

Following the completion of the initial 4 sessions (month 1), a follow-up was conducted in the intervention group through personal telephone interviews, totalling 6 calls: 2 calls in the second month (1 caLL every 2 weeks) and 1 caLL per month from the third to the sixth month. During these follow-up calls, participants were asked about their adherence to the provided recommendations, and they were encouraged to maintain those healthy lifestyles. Any doubts or concerns were addressed, and personalized solutions were provided when necessary.

### 2.4 Outcomes

#### 2.4.1 Frailty phenotype

Fried’s criteria stratify older adults as robust, pre-frail, or frail based on the final score of the following domains (17): shrinking (participants score a point if they had an unintentional weight loss of 4.5 kg or 5% in the previous year), weakness (if their grip strength was less than 29 kg when their BMI was equal to or below 24, 30 kg when their BMI fell within the range of 24.1 to 28, and 32 kg if their BMI exceeded 28 for men. For women, performance below the threshold was defined as grip strength less than 17.1 kg when their BMI was equal to or below 23, 17.3 kg for BMI values between 23.1 and 26, 18 kg for BMI values between 26.1 and 29, and 21 kg if their BMI exceeded 29, T.K.K.5401 GRIP D, Takei Scientific Instruments Co., Ltd., Japan), poor endurance (if they reported self-reported exhaustion or fatigue for 3 days or more in the past week), slowness [if they performed the 4-meter walk in 7 s or more (men height ≤ 173 cm, women height ≤ 159 cm) or 6 s or more (men height > 173 cm, women height > 159 cm)], and low physical activity (reported reduced physical activity expenditure). The classification of participants was based on specific criteria: those who did not meet any criteria were categorized as robust, individuals meeting one or two of the criteria were classified as pre-frail, and those who presented three or more criteria were designated as frail.

#### 2.4.2 Physical function

The participants’ physical function was assessed using the Short Physical Performance Battery (SPPB), a validated tool specifically designed for older individuals (18). The SPPB evaluates three key physical domains: (i) balance, which consists of three different tests (side-by-side, semi-tandem, and tandem tests), (ii) gait speed, evaluated with the 4-meter gait test, and (iii) a functional measure of lower-limb strength, assessed through the 5-repetition sit-to-stand test. Each domain is scored based on participants’ performance with scores ranging from 0 to 4 points. Participants who were unable to complete a specific test were assigned a score of 0. The final SPPB score reflects overall physical function and ranges from 0 to 12, where 0 indicates the highest dependence and limitations in mobility, and 12 indicates complete independence in physical tasks. In addition to the SPPB evaluation, the 5-repetition sit-to-stand test power was calculated using the formula proposed by Baltasar-Fernandez et al. (19).

#### 2.4.3 Physical activity, inactivity, and sleep behaviour

Physical activity, inactivity, and sleep behaviour were assessed using wrist-worn accelerometers (GENEActiv, ActivInsights Ltd., Kimbolton, UK). To ensure accurate and reliable measurements, participants were instructed to wear the accelerometer on their non-dominant wrist continuously for a minimum of six consecutive days. Only data from participants who wore the accelerometer for at least 16 h per day on a minimum of four days, including at least three weekdays and one weekend day, were considered valid and included in the analysis. The accelerometers were set to record data at a frequency of 60 Hz, and the raw data were downloaded using GENEActiv software version 3.3. Subsequently, the raw data files were processed and analysed using the open-source R-package GGIR, version 2.5–0, on the University of Malaga servers. The GGIR package automatically calibrated the data based on local gravity and calculated the Euclidean Norm Minus One (ENMO) for accelerations, helping to minimize sensor calibration errors (20). Given the fact that wrist-worn accelerometers cannot detect lower body posture, and therefore cannot detect if the lower intensity is made in a standing, reclining, or sitting position which is essential to determine sedentary behaviour, the lower intensity has been defined as inactivity (21).

To classify participants’ activity levels, previously established thresholds for ENMO in the wrist of older adults were applied (22, 23). The following intensity categories were used: (i) Inactivity: ENMO ≤57 milligravities (mG), (ii) Light-intensity Physical Activity (LPA): ENMO >57 mG and < 104 mG, (iii) Moderate-to-Vigorous-intensity Physical Activity (MVPA): ENMO ≥104 mG. Physical activity and inactivity were quantified as mean minutes per day, calculated by dividing the total minutes spent in each respective category by the total number of valid days.

Regarding sleep behaviour, inactivity periods with low variability in the z angle (less than 5 degrees over 5 min) were identified as sustained inactivity periods. An automated algorithm, based on the work by van Hees et al. (24), was employed to detect the onset and offset of sleep periods using these sustained inactivity periods during the night. Time in bed was defined as the duration between when participants lay down with low variability in the z angle (less than 5 degrees over 5 min) and their wake-up time during the day. Sleep time was estimated by calculating the difference between sleep onset and wake-up time. Wake After Sleep Onset (WASO) was determined as the total duration of wakefulness between sleep onset and sleep termination. Awakenings were identified as instances of wakefulness lasting at least 5 min during the sleep period (24), and the number of awakenings per night was recorded. Lastly, sleep efficiency was determined as the proportion of time spent sleeping from sleep onset to sleep termination, taking into account the duration of wakefulness (WASO). Sleep efficiency is expressed as a percentage, where a score of 100 is the best possible result, signifying uninterrupted sleep from sleep onset to termination without any periods of wakefulness.

#### 2.4.4 Nutritional assessment

Participants’ body mass was assessed using a digital scale (Omron Medizintechnik, Mannheim, Germany), with any footwear, heavy clothing, and accessories removed. Body height was measured with participants standing on the Frankfort plane, after exhaling normally, utilizing a stature-measuring instrument (SECA 225, Hamburg, Germany). Body Mass Index (BMI) was calculated by dividing the body mass (in kilograms) by the square of the height (in meters). Waist, left arm, and left leg circumferences were measured using a metallic non-extensible tape (Lufkin W606PM, Washington, United States) following the International Standards for Anthropometric Assessment (ISAK) guidelines standard techniques by an ISAK level 1 evaluator. The waist circumference was measured at the thinnest point, while the arm and leg circumferences were measured at the most prominent points. All measurements were conducted twice by an experienced examiner, and the final value was obtained as the mean between the two measurements.

Moreover, the Mini Nutritional Assessment (MNA) was applied through a personal interview to screen for the risk of malnutrition among the participants (18). The overall MNA score can differentiate between older patients with adequate nutritional status (MNA score ≥ 24), with a risk of malnutrition (MNA score between 17 and 23.5), or with protein-calorie undernutrition (MNA score ≤ 17). The sensitivity, specificity, and diagnostic accuracy of the MNA questionnaire were 96%, 98%, and 97%, respectively (25).

### 2.5 Sample size and statistical analysis

The sample size calculation was performed using the statistical software Epidat 4.2. It was calculated to detect the smallest significant effects on the main variable. Assuming a large intervention effect using an F test with two tails (0.5), an alpha error of 0.05, 80% power, and accounting for a dropout rate of 10%, it was determined that two groups of 80 subjects each would be required. Additionally, after evaluating the data using the G*Power software (v. 3.1.9.7, Universität Kiel, Germany), we found an actual power of 0.96 in one of our main outcomes, specifically the SPPB score.

To maintain the initial comparability between the groups, and to preserve the sample size while minimizing bias, an intention-to-treat analysis was employed. All data are presented as mean ± standard deviation (SD) or counts (percentages). The normality of continuous variables was assessed using the Kolmogorov–Smirnov test, the Levene test was used to check the homogeneity of variance and sphericity with the Mauchly test. A 2×2 mixed factorial analysis of variance (ANOVA) was used, with the group (intervention vs. control) as the between-subjects factor, and the time (pre-test vs. post-test) as the within-subject factor. Bonferroni post-hoc comparisons were applied. The effect size of each variable was tested using partial eta squared (η2p), with values of 0.01, 0.06, and 0.14 used to indicate small, medium, and large effects, respectively. After carefully examining potential confounding factors, we included sex, age, frailty phenotype, BMI and educational level as covariates in the analysis. Deltas, representing the changes from post-intervention values to post-intervention values, were calculated for the key parameters as post-intervention values minus pre-intervention values. These changes were then compared between groups using either an independent sample t-test or a Mann–Whitney U-test, depending on the normality distribution of the variables. Furthermore, an Quade’s analysis of covariance (Quade’s ANCOVA) was conducted, employing the same covariates as in the previous analysis. All statistical analyses were performed using IBM SPSS Statistics version 26 software (SPSS Inc., Chicago, IL, United States) and STATA 13.0 (StataCorp LLC, Texas, United States). The significance level was set at *p* < 0.05 to determine statistical significance.

## 3 Results

Table 1 displays the sociodemographic and medical characteristics of our participants. It’s noteworthy that the intervention group had a higher proportion of women, was characterized by older age, and exhibited a higher level of education. Due to these differences, we have decided to include these variables as covariates in the forthcoming analysis.

Table 2 presents the frailty characteristics of the study participants. In the baseline assessment, the intervention group exhibited lower scores on Fried’s criteria and a higher number of pre-frail participants compared to the control group. Additionally, a larger proportion of participants in the control group met the criteria for adequate physical activity (*p* = 0.016) and handgrip strength (*p* < 0.001) compared to the intervention group. Following the intervention, there were no significant changes observed in the control group regarding total frailty points or specific criteria. However, a noteworthy reduction in total frailty points was observed in the intervention group (*p* < 0.001), along with a decrease in the number of participants meeting the self-reported exhaustion and fatigue criteria (*p* < 0.002) and a decrease in the prevalence of individuals with low handgrip strength (*p* < 0.001).

**Table 2:** Comparison of participant frailty outcomes before and after the intervention.

	Control group (83)		Educational group (80)	
	PRE	POST	PRE	POST
Total fried (points, median [IQR])	2.0 [2.0–3.0]	2.0 [1.0–3.0]	2.0 [1.0–2.0]†	1.0 [0.0–2.0]*†
Fried’s category, *n* (%)				
Robust	0 (0)	0 (0)	0 (0)	24 (30)*†
Pre-frail	45 (54,2)	43 (51.8)	65 (81.3)†	50 (62.5)*†
Frail	38 (45,8)	40 (48.2)	15 (18.8)†	6 (7.5)*
Unintended weight loss, *n* (%)				
Lost <5% of body mass	52 (62.7)	69 (83.1)	57 (71.3)	68 (85)*
Lost >5% of body mass	31 (37.3)	14 (16.9)	23 (28.8)	12 (15)*
Self-reported exhaustion and fatigue, *n* (%)				
Did not meet the criteria	23 (27.7)	21 (25.3)	20 (25)	40 (50)*†
Met the criteria	60 (72.3)	62 (74.7)	60 (75)	40 (50)*†
Physical activity expenditure, *n* (%)				
Low	14 (16.9)	19 (22.9)	4 (5)†	11 (13.8)
Normal	69 (83.1)	63 (75.9)	76 (95)†	69 (86.3)*
Gait speed, *n* (%)				
Low	28 (33.7)	38 (45.8)	22 (27.5)	13 (16.3)†
Normal	55 (66.3)	45 (54.2)	58 (72.5)	67 (83.8)†
Handgrip strength, *n* (%)				
Low	69 (83.1)	69 (83.1)	39 (48.8)	20 (25)*
Normal	14 (16.9)	14 (16.9)	41 (51.3)	60 (75)*

IQR, interquartile range[percentile 25 – percentile 75]. * *p* < 0.05 compared to the pre-intervention moment within the same group; † *p* < 0.05 compared to the control group at the same moment.

**Figure 2:**
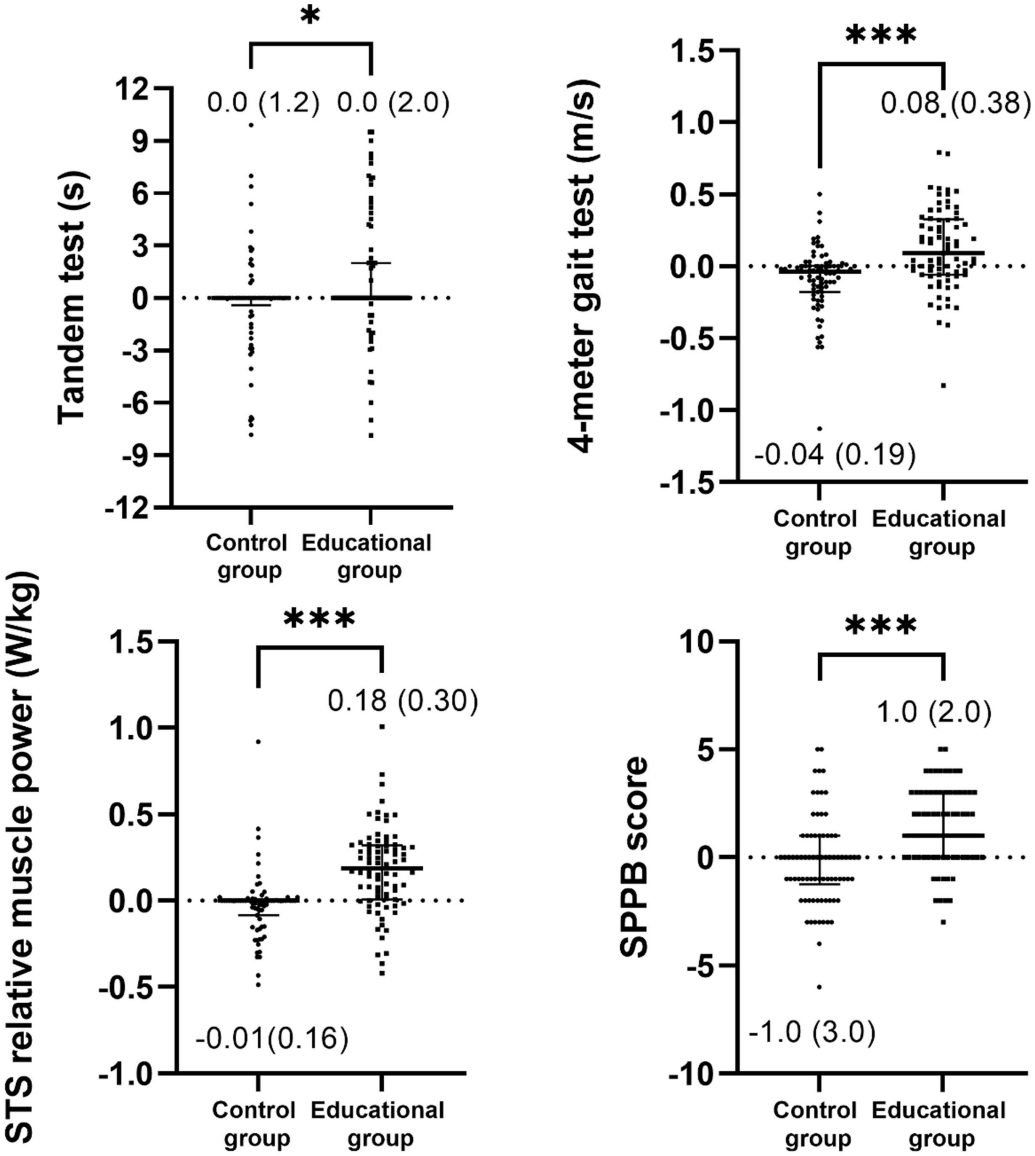
Comparison of post-intervention minus pre-intervention delta values for physical function between the control and educational groups (unadjusted). Data is presented as median (interquartile range). STS, sit-to-stand test, and SPPB, short physical performance battery. Statistical significance is indicated by asterisks: *p < 0.05, **p < 0.01, ***p < 0.001.

**Figure 3:**
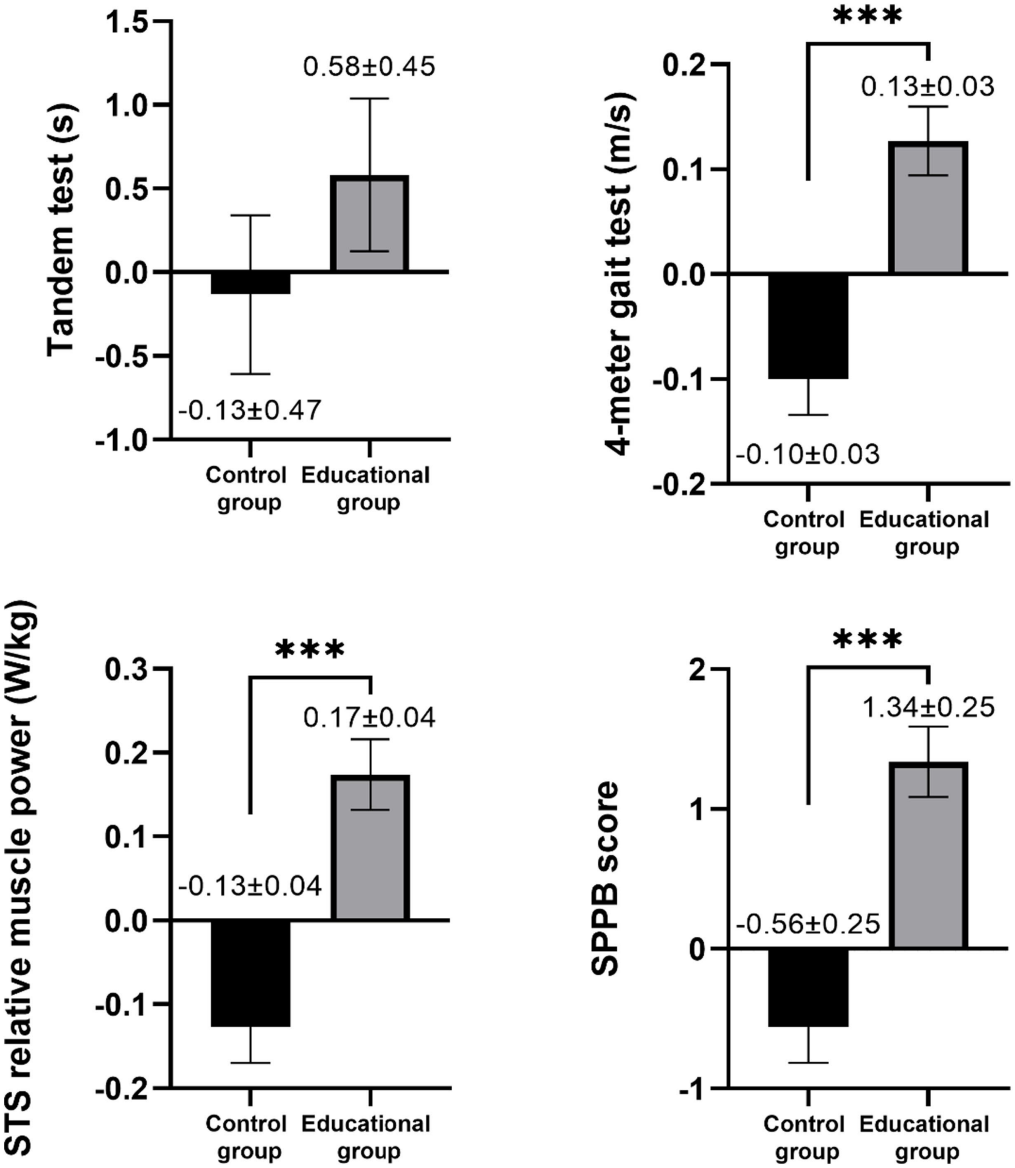
Adjusted comparison of post-intervention minus pre-intervention delta values for physical function between the control and educational groups. Data is displayed as the mean ± standard error of the mean. STS, sit-to-stand test, and SPPB, short physical performance battery. Statistical significance is indicated by asterisks: *p < 0.05, **p < 0.01, ***p < 0.001.

All mean values of the outcomes before and after the intervention are presented in Table 3 with a comparison between groups in both the pre- and post-intervention. Regarding physical function, a significant group x time interaction was observed in the SPPB score (*F* = 35.043, η2p = 0.179, *p* < 0.001), the 4-meter gait test (*F* = 22.503, η2p = 0.122, *p* < 0.001), the 5-repetition sit-to-stand test (*F* = 11.316, η2p = 0.066, *p* < 0.001) and the 5-repetition sit-to-stand power (*F* = 24.994, η2p = 0.136, *p* < 0.001). When the delta values were compared, the results demonstrated a significant improvement in physical function in the intervention group in comparison to the control group (Figure 2). After adjusting for covariables, significant differences persisted in side-by-side (*p* < 0.001), semi-tandem (*p* < 0.001), tandem (*p* < 0.001), as well as the SPPB score (*p* = 0.001) and the SST power (*p* < 0.001) while the differences in gait test and five-repetition sit-to-stand test disappeared. In the post-intervention assessment, these differences continued to be significant (*p* < 0.001). Furthermore, the 5-repetition sit-to-stand power exhibited significant differences between the groups (*p* < 0.001). Subsequent Bonferroni *post hoc* comparisons revealed a significant decrease in the SPPB score for the control group (*p* = 0.042) but a significant improvement in the gait speed (*p* = 0.004), sit-to-stand test (*p* = 0.001), SPPB score (*p* < 0.001), and SST power in the intervention group (Supplementary Table S1). When the delta values were compared using the ANCOVA, it revealed an enhanced physical function in the intervention group, with the exception of the tandem test, which no longer exhibited statistical significance (Figure 3).

**Table 3:** Comparison of participant outcomes before and after the intervention.

	Control group (83)		Educational group (80)	
	PRE	POST	PRE	POST
Physical function				
Side-by-side test (s, median [IQR])	10.0 [0.7–10.0]	10.0 [7.1–10.0]*	10.0 [10.0–10.0]†	10.0 [10.0–10.0]†
Semi tandem test (s, median [IQR])	10.0 [0.0–10.0]	10.0 [0.0–10.0]	10.0 [10.0–10.0]†	10.0 [10.0–10.0]†
Tandem test (s, median [IQR])	3.2 [0.0–10.0]	3.1 [0.0–10.0]	10.0 [4.5–10.0]†	10.0 [8.0–10.0]*†
4-meter gait test (s, median [IQR])	5.3 [4.5–7.3]	6.2 [4.8–10.1]*	4.9 [4.1–6.0]†	4.2 [4.0–5.7]*†
Five-repetition sit-to-stand test (s, median [IQR])	15.0 [12.0–40.0]	17.7 [12.9–39.9]	15.3 [11.9–19.9]†	11.1 [9.6–13.8]*†
SPPB score (points, median [IQR])	7.0 [4.8–9.0]	6.0 [4.0–8.0]*	9.0 [8.0–10.0]†	10.0 [9.0–12.0]*†
Handgrip strength (kg, median [IQR])	20.0 [13.6–26.9]	20.0 [12.5–26.6]	20.6 [17.3–27.2]†	20.1 [16.6–26.3]
STS relative muscle power (W/kg, median [IQR])	0.6 [0.2–0.8]	0.5 [0.2–0.6]	0.6 [0.4–0.8]	0.8 [0.6–1.0]*†
Physical activity				
Inactitivy (min/day, mean ± sd)	802.26 ± 139.74	810.4 ± 131.97	829.31 ± 110.43	836.49 ± 104.27
MVPA (min/day, median [IQR])	0.0 [0.0–11.3]	3.2 [0.0–22.5]	1.7 [0.0–8.3]	1.7 [0.0–9.1]
LPA (min/day, mean ± sd)	116.24 ± 140.34	103.27 ± 62.53	98.98 ± 47.21	91.67 ± 47.14
Sleep behaviour				
Bedtime (h/day, mean ± sd)	7.69 ± 1.48	8.21 ± 1.80	7.71 ± 1.53	7.97 ± 1.44
Sleep time (h/day, mean ± sd)	6.38 ± 1.46	6.82 ± 1.67	6.66 ± 1.41	6.94 ± 1.32
Awakenings (number/day, mean ± sd)	12.98 ± 4.31	14.30 ± 4.55*	13.44 ± 4.27	13.33 ± 4.42
Sleep efficiency (%, median [IQR])	86.6 [82.2–89.9]	85.3 [81.1–88.7]	84.8 [81.1–91.0]	87.8 [81.1–91.0]
Nutritional assessment				
IMC (kg/m2, mean ± sd)	28.85 ± 4.82	29.01 ± 5.19	29.84 ± 4.79	29.81 ± 4.94
Waist perimeter (cm, mean ± sd)	100.48 ± 13.94	100.15 ± 14.69	99.33 ± 11.08	97.97 ± 10.42
Arm perimeter (cm, mean ± sd)	29.31 ± 3.89	28.39 ± 3.73	28.86 ± 3.79	28.65 ± 3.99
Leg perimeter (cm, median [IQR])	35.0 [32.0–38.0]	35.0 [32.0–38.0]	35.5 [32.0–37.8]	35.1 [32.5–37.6]
MNA (score, median [IQR])	26.5 [25.0–27.9]	25.5 [23.0–26.5]*	25.0 [22.0–26.8]	26.0 [24.0–27.8]*†

IQR, interquartile range [percentile 25 – percentile 75]; sd, standard deviation, STS, sit-to-stand test; SPPB, Short physical performance battery; MVPA, moderate to vigorous physical activity; LPA, light physical activity; BMI, body mass index; MNA, mini nutritional assessment. Asterisks (*) indicate *p* < 0.05 compared to the pre-intervention values within the same group, and daggers (†) indicate *p* < 0.05 compared to the control group at the same time point.

**Figure 4:**
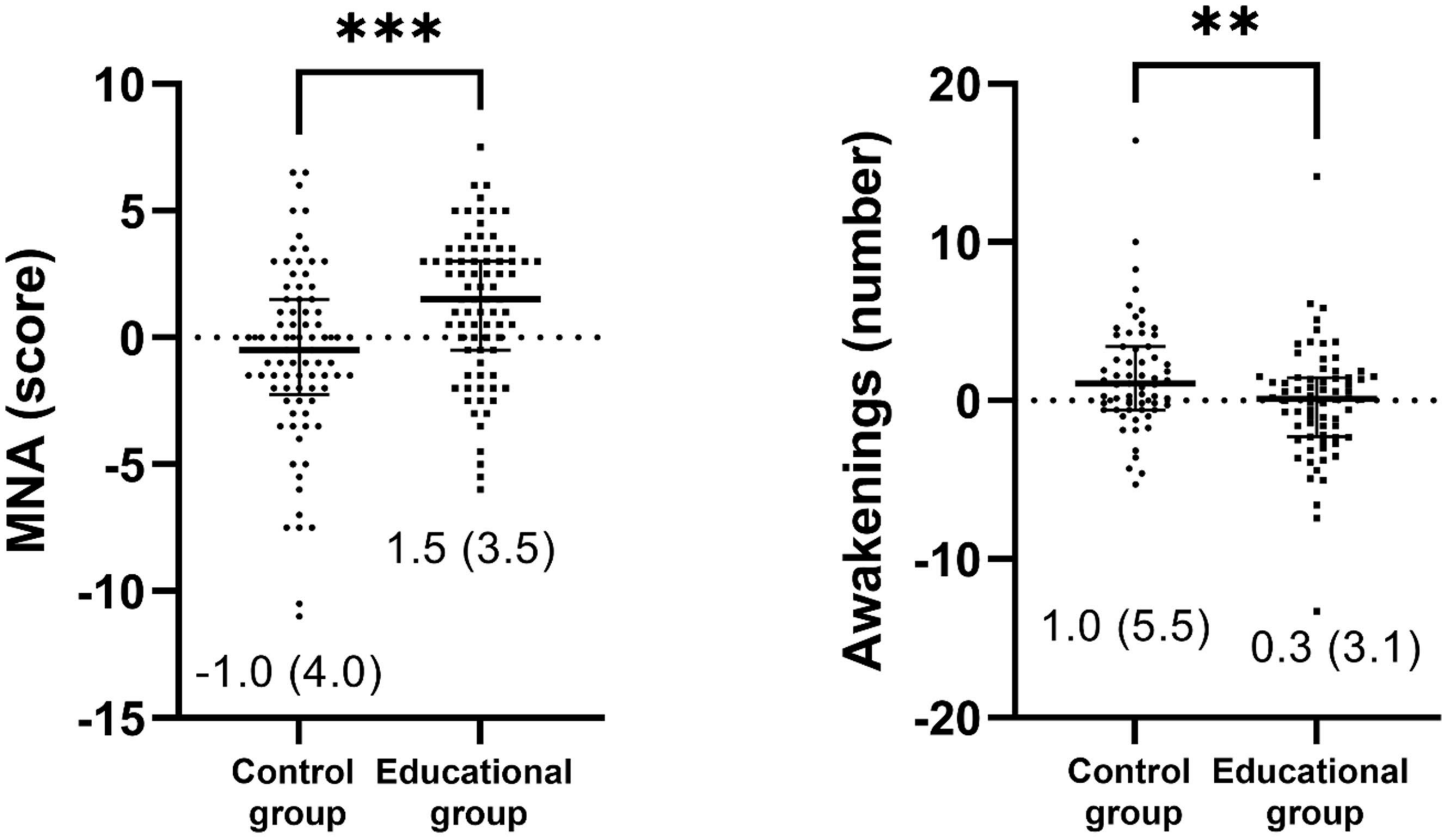
Comparison of post-intervention minus pre-intervention delta values of sleep and nutritional assessment for the control group and educational group (unadjusted). Data is presented as median (interquartile range). MNA, mini nutritional assessment, *p < 0.05, **p < 0.01, ***p < 0.001.

**Figure 5:**
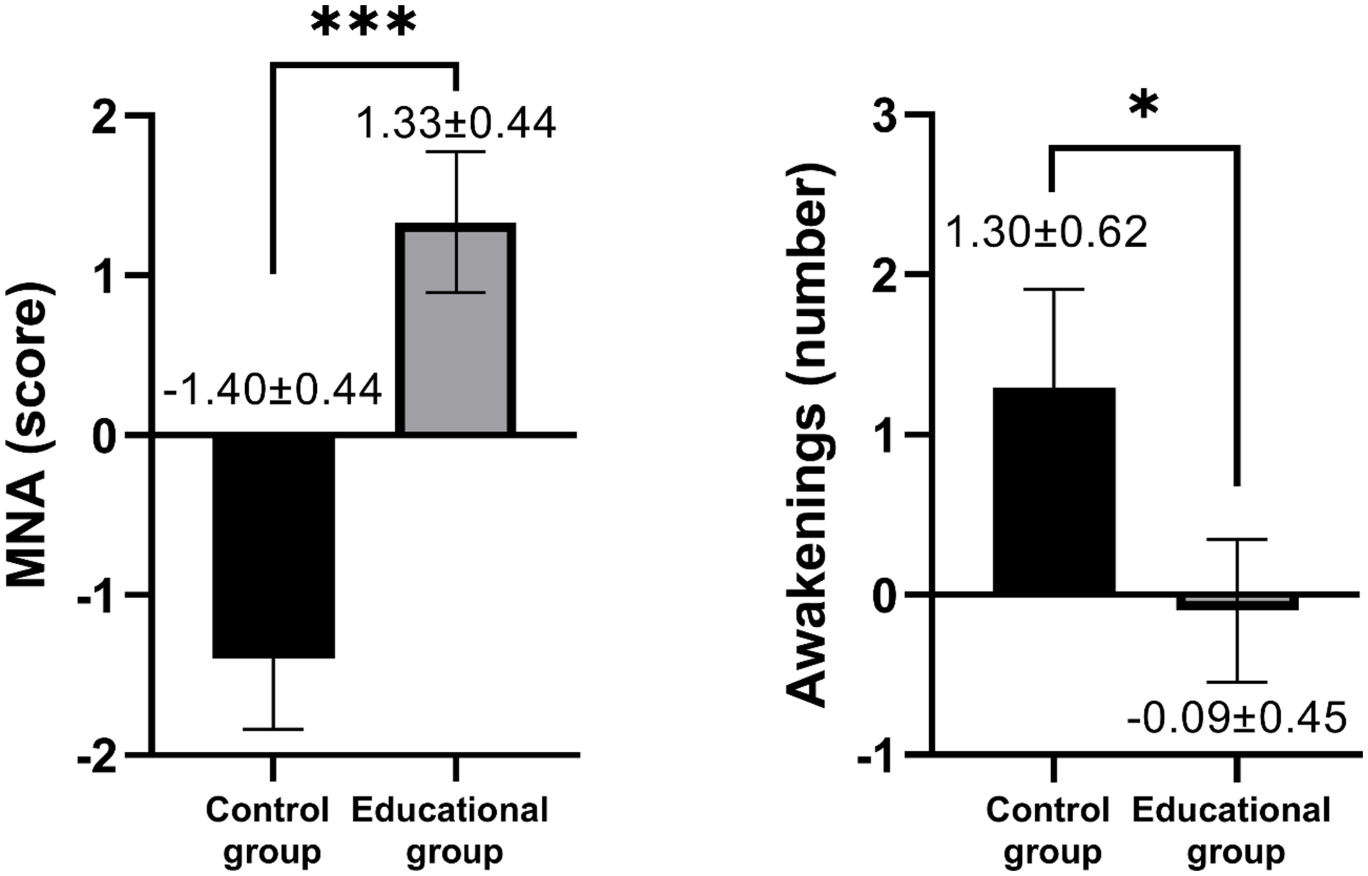
Adjusted comparison of post-intervention minus pre-intervention delta values for sleep and nutritional assessment between the control and educational groups. Data is displayed as the mean ± standard error of the mean. MNA, mini nutritional assessment. Statistical significance is indicated by asterisks: *p < 0.05, **p < 0.01, ***p < 0.001.

The accelerometer analyses showed a significant group x time interaction in the number of awakenings at night (*F* = 5.610, η2p = 0.040, *p* = 0.019), indicating an increase in the number of awakenings in the control group, while the educational group maintained similar values from pre- to post-intervention (Figure 4). However, no significant group x time interaction was found in physical activity and inactivity values, even after adjusting for confounders (Supplementary Table S1). When the delta values were compared using the ANCOVA, the results remained significant (Figure 5).

Regarding the nutritional status, there was a significant group x time interaction in the MNA score (*F* = 15.737, η2p = 0.088, *p* < 0.001). Specifically, the educational group showed an improvement in MNA score from pre- to post-intervention, while the control group exhibited a significant decrease in the MNA score (Figure 4). Nevertheless, there were no differences between groups on perimeters, body mass or BMI even after adjusting by cofounders. When the delta values were compared using the ANCOVA, the results remained significant (Figure 5).

## 4 Discussion

The main findings of this study underscore that older adults with pre-frailty or frailty who underwent a 6-month educational intervention experienced enhancements in their frailty outcomes, physical function, sleep patterns, and nutritional status when compared to the control group. These results highlight the potential of educational interventions in promoting a healthy ageing approach for this vulnerable population.

Frailty is a significant concern in healthcare due to its impact on the longevity and quality of life of older populations (26). Importantly, frailty is a reversible condition through various interventions. However, there is ongoing controversy in the literature regarding the effectiveness of multifaceted programs in reversing frailty among community-dwelling older individuals. Some studies have reported beneficial results (27, 28), while others have not (29). The reason for these differences could be attributed to the varying definitions of frailty used in the studies, such as Fried’s criteria (17), the Frail Trait Scale (30) or other frailty indicators (31). The results of our study contribute additional insights to this topic. We found that 30% of participants in the intervention group no longer met Fried’s criteria for frailty after participating in the FRAGSALUD program for six months, which consisted of attending just four group sessions. This suggests that multi-targeted programs encompassing frailty, physical exercise, nutrition, and cognition could be an effective strategy in improving the frailty status of older adults, especially self-reported exhaustion and fatigue and the cut-off point for handgrip strength. Nonetheless, it is worth mentioning that the effectiveness of this program remains to be seen when using other valid tools to evaluate frailty (30).

It is known that impaired physical function is a major indicator of frailty, compromising healthy ageing (32). Our results highlight the effectiveness of the educational intervention to enhance physical function among frail/pre-frail older adults. Specifically, a large effect in the SPPB score and moderate effect sizes in the 4-meter gait test, and the 5-repetition sit-to-stand test performance and power were found. Notably, the educational intervention led to a significant improvement of 1.49 points in the SPBB score, surpassing the threshold for clinically meaningful change (typically set at a minimum of 0.5 points) (33). It is noteworthy that minimal clinically important improvements in gait speed have been reported to be 0.06 m/s (34), and in our study, an increase of 0.07 m/s was achieved in the 4-meter gait test in the educational group, surpassing this threshold. Similarly, previous studies have reported that lifestyle educational programs conducted over 24 months improved physical function with an increase of 1.2 points on the SPPB score (35, 36), while a multicomponent intervention over 3 months led to a 1-point increase (37) in frail older adults. Also, interventions targeting nutrition, cognition, and physical activity have shown decreases in gait speed completion times, such as 0.8 s, 1.10 s, and 0.54 s, respectively, over 6 months in pre-frail and frail older populations (38). It is worth noting that specific exercise programs have been shown to result in a larger increase in SPPB scores, with improvements of around 3 points (39, 40). However, these interventions typically involved a structured regimen of between 18 and 72 exercise-focused sessions. The FRAGSALUD intervention achieved almost half the improvement with just 4 educational sessions, which shows a promising result in cost-effective intervention for healthcare. It’s important to highlight that these effects remained statistically significant even after adjustments for potential covariates such as sex, age, physical frailty status, BMI, and educational status. Given that poor physical performance has been demonstrated to be more common among older individuals, women, and those with lower levels of education (41), the sustained significance of these results after adjusting for these covariates suggests that the FRAGSALUD study represents an effective intervention for older adults residing in the community, irrespective of their age, sex, or educational status.

Two systematic reviews have indicated that exercise and nutritional interventions may not be effective in improving the results of the tandem test and the sit-to-stand test in pre-frail and frail adults (42). In contrast, the educational program conducted in the FRAGSALUD project achieved improvements in balance and lower body performance in community-dwelling older adults with frailty or pre-frailty. However, it is possible that even though there are no predefined cut-off points for balance (34) and lower-limb strength performance (43) in older adults, the improvements observed in the educational program in these tests are still beneficial for the older population. In this regard, the FRAGSALUD program achieved a reduction of nearly 8 s in the performance time of the 5-repetition sit-to-stand test and an improvement in the 5-repetition sit-to-stand test power by 28%. While this improvement is slightly lower than the 47% increase reported in a six-week exercise-focused program (39), it is still noteworthy that in only 4 sessions, the muscle power of older adults with frailty phenotype increased almost 30%. However, it’s noteworthy that the improvements observed in the tandem test disappeared when the analysis was adjusted for covariates. This implies that these improvements may be more closely related to factors such as age, sex, frailty status, or educational background rather than the intervention itself.

Despite the significant improvements in physical function, our findings were not directly attributed to the levels of physical activity or inactivity measured by accelerometry over one week, neither in the unadjusted models nor in the adjusted models. Both the control and intervention groups maintained similar physical activity levels after 6 months. These results align with findings from previous studies (44). Indeed, a systematic review has revealed that approximately half of the health education interventions designed to enhance physical activity were not successful (45). This suggests that such interventions may not be effective in increasing physical activity in this particular population. In addition to this, the results from this study could be explained due to the device used. It is crucial to acknowledge that the accelerometers employed in this study were primarily designed to assess ambulatory physical activity and may not accurately capture the stationary resistance exercises that were incorporated into our program (46). Indeed, positive health effects associated with increased MVPA have been well-documented in this population according to previous studies (47). Thus, this limitation of accelerometers in capturing resistance exercises could potentially explain the lack of a significant intervention effect on physical activity outcomes.

Previous studies have demonstrated that frailty is associated with a significant decrease in sleep quality (48). Moreover, as individuals age, notable changes occur in sleep patterns, including alterations in sleep quality and quantity, such as slow-wave sleep, spindle density, and sleep continuity (49). However, the existing literature on interventions to enhance sleep in older adults remains limited, with exercise being the primary consideration for potential improvement in sleep quality for this population (50). Similarly, maintaining adequate nutrition has been shown to improve the sleep quality of older adults (51). Consequently, given that two of the sessions of the FRAGSALUD program were focused on both exercise and nutrition, an improvement in sleep quality or efficiency was expected. Nonetheless, no significant changes were found for almost any of the sleep variables assessed with both groups obtaining similar results in both the pre- and post-intervention both in the unadjusted models nor in the adjusted models. This could be due to sleep being measured using a wrist-worn accelerometer which could be seen as a limitation due to the accelerometer estimating sleep from movement patterns and not asses the actual sleep. Notwithstanding, sleep patterns assessed through accelerometry showed that the nocturnal awakenings were prevented in the intervention group of the FRAGSALUD study. Notably, the control group experienced a noteworthy increase of 1.32 awakenings per night after 6 months, while the educational group maintained stable levels of awakenings. Despite the increase in the number of awakenings in the control group, it is worth mentioning that this did not have a noticeable effect on sleep efficiency, which might compromise the use of this measure (i.e., sleep efficiency) for establishing recommendations for healthy ageing in frail older adults.

Nutritional habits and nutritional status are also essential factors in promoting healthy ageing and hold special relevance in the context of frailty (52). A qualitative assessment of nutritional status using MNA has been associated not only with the progression of frailty (53) but also with a lower risk of developing cognitive frailty in individuals who meet at least one of the criteria for this condition (16). Moderate effect sizes were observed in the MNA scores of this population, with similar improvements reported by previous studies with physical training and nutritional interventions conducted in older adults with pre-frailty and frailty (54). Nutrition, either alone or in combination with exercise, plays a significant role in managing malnutrition (55). Previous studies have demonstrated the effectiveness of exercise and nutritional education in improving the frailty status of community-dwelling adults aged 60 years or older (9). The results of this study emphasize the potential benefits of educational interventions in improving nutritional status in community-dwelling older adults and potentially mitigating the adverse consequences linked to frailty, irrespective of their age, sex, physical frailty status, and educational background. This is an interesting result, particularly given the association of malnutrition with age (56). The observed improvement, irrespective of the participants’ age or other potential covariates such as educational status (which could be linked to a better understanding of the intervention), indicates that the FRAGSALUD intervention is an effective program for reducing the risk of malnutrition in older adults living in the community. However, despite the reduction in the risk of malnutrition in the intervention group, no significant differences were found for any of the anthropometric variables in the present study.

This study has several limitations that should be considered. Firstly, the study’s sample was limited to participants from the southern region of Spain, potentially restricting the generalizability of the findings to other populations or geographic areas. Furthermore, it’s crucial to note that the study’s results only reflect the outcomes of the educational intervention at the 6-month mark. To gain a more comprehensive understanding of the long-term effects and sustainability of these changes, conducting follow-up evaluations over an extended period would be valuable. Future studies should investigate whether these changes endure over time. Furthermore, it’s important to acknowledge that the control group was older and had more Fried’s criteria than the intervention group at the outset, which could potentially influence the results. However, it’s noteworthy that after six months, the results in the control group remained consistent, as well as the results after adjustin by these covariates This suggests that the differences observed between the two groups were not solely due to age or baseline frailty criteria but were likely influenced by the intervention itself. Nonetheless, despite the inclusion of some covariates in the analysis, it’s worth noting that other factors, such as economic status, which were not included, may also impact the results. This represents a limitation of the study. Another limitation relates to the assessment of malnutrition risk, which relied on interviewer questionnaires. This approach is susceptible to recall bias and may not accurately reflect participants’ actual behaviours. Additionally, the accelerometer used in the study was worn on the wrist and assessed sleep based on movement patterns rather than directly measuring sleep. Nonetheless, wrist-worn accelerometers are generally considered a minimally invasive method with good validity for assessing sleep behaviours in day-to-day life (57). Lastly, the study did not incorporate motivational interviews to change behaviour, which could potentially limit the impact of the program.

Nonetheless, this study has several strengths. The FRAGSALUD program is an intervention easy to implement in the healthcare system, based on only 4 multitargeted sessions with phone calls as follow-up control during the subsequent months, making it an easy and useful tool for the healthcare system. In addition to this, frailty has been evaluated using different tools such as Fried’s questionnaire which focuses on both physical function and mental state, and SPPB evaluation which mainly focuses on physical frailty. Similarly, this study includes an integral evaluation of older adults, encompassing physical activity and sleep using more objective tools than questionnaires, nutritional assessment, physical function, and frailty.

## 5 Conclusion

The 6-month educational program improved physical function, sleep patterns, and nutritional status compared to the usual healthcare attendance in community-dwelling older adults with frailty or pre-frailty, regardless of their sex, age, frailty status, and educational background. The educational intervention of the FRAGSALUD study, consisting of 4 grouped sessions with 6 follow-up calls over 6 months, not only proved to be effective but also minimized associated challenges, making it easily implementable within care routines. The simplicity and affordability of the FRAGSALUD intervention make it a feasible option for addressing frailty in older adults living in the community, potentially contributing to healthy ageing. Further studies with a longer follow-up are encouraged.

## Data availability statement

The raw data supporting the conclusions of this article will be made available by the authors, without undue reservation.

## Ethics statement

The studies involving humans were approved by the Committee of the Provincial Research of Málaga (Approval code: FRAGSALUD). The studies were conducted in accordance with the local legislation and institutional requirements. The participants provided their written informed consent to participate in this study.

## Author contributions

CC: Conceptualization, Funding acquisition, Methodology, Writing – original draft. LÁ-C-d-V: Investigation, Writing – review & editing. AG-M: Investigation, Writing – review & editing. AM-G: Investigation, Writing – review & editing. MC: Investigation, Writing – review & editing. JP-G: Funding acquisition, Writing – review & editing. MV-S: Conceptualization, Funding acquisition, Methodology, Writing – original draft. JC-P: Funding acquisition, Methodology, Writing – original draft.

## Footnotes

^1^ http://www.randomization.com
