# Supplementary material for: Effects of an educational intervention on frailty status, physical function, physical activity, sleep patterns, and nutritional status of older adults with frailty or pre-frailty: the FRAGSALUD study
Source: Front Public Health. 2023 Nov 30;11:1267666. doi: 10.3389/fpubh.2023.1267666 (PMC10720710; doi:10.3389/fpubh.2023.1267666)
Supplement: Supplementary file 1 [file Table_1.DOCX]

| **Supplementary Table 1. Comparison of Participant Outcomes Before and After the Intervention after adjusting by covariates** | | | | |
| --- | --- | --- | --- | --- |
|  | **Control Group (83)** | | **Educational Group (80)** | |
|  | **PRE** | **POST** | **PRE** | **POST** |
| ***Physical Function*** | | | | |
| Side-bySide test (s) | 6.89 ± 0.37 | 8.01 ± 0.32* | 9.86 ± 0.36† | 9.79 ± 0.31† |
| Semi tandem test (s) | 6.57 ± 0.39 | 6.59 ± 0.38 | 9.34 ± 0.39† | 9.58 ± 0.36† |
| Tandem test (s) | 5.05 ± 0.48 | 4.80 ± 0.42 | 7.44 ± 0.45† | 8.12 ± 0.41† |
| 4-meter gait test (s) | 5.78 ± 0.25 | 7.07 ± 0.29* | 5.45 ± 0.23 | 4.46 ± 0.35*† |
| Five-repetition sit-to-stand test (s) | 22.69 ± 2.03 | 28.51 ± 1.84* | 20.83 ± 1.96 | 13.65 ± 1.76*† |
| SPPB score | 7.28 ± 0.25 | 6.58 ± 0.26* | 8.44 ± 0.25† | 9.91 ± 0.25*† |
| Handgrip strength (kg) | 22.54 ± 1.04 | 22.24 ± 1.00 | 20.38 ± 1.01 | 20.42 ± 0.96 |
| STS relative muscle power (W/kg) | 0.75 ± 0.04 | 0.60 ± 0.04* | 0.57 ± 0.05† | 0.77 ± 0.03*† |
| ***Physical Activity*** | | | | |
| Inactitivy (min/day) | 775.35 ± 25.31 | 771.96 ± 22.44 | 832.42 ± 17.02 | 852.42 ± 15.01 |
| MVPA (min/day) | 8.18 ± 2.53 | 12.55 ± 2.66 | 9.28 ± 2.15 | 8.84 ± 2.26 |
| LPA (min/day) | 137.41 ± 20.72 | 116.35 ± 11.29 | 106.29 ± 13.93 | 92.66 ± 7.59 |
| ***Sleep behaviour*** | | | | |
| Bedtime (h/day) | 7.72 ± 0.23 | 8.23 ± 0.26 | 7.67 ± 0.17 | 7.83 ± 0.19 |
| Sleep time (h/day) | 6.59 ± 0.19 | 6.90 ± 0.23 | 6.63 ± 0.15 | 6.82 ± 0.17 |
| Awakenings (number/day) | 13.20 ± 0.71 | 14.63 ± 0.52* | 13.23 ± 0.52 | 13.06 ± 0.54 |
| Sleep efficiency (%) | 83.80 ± 0.01 | 83.70 ± 0.01 | 86.1 ± 0.01 | 0.86 ± 0.10 |
| ***Nutritional assessment*** | | | | |
| IMC (kg/m2) | 29.18 ± 0.56 | 29.29 ± 0.59 | 29.71 ± 0.56 | 29.69 ± 0.59 |
| Waist perimeter (cm) | 102.04 ± 0.98 | 101.23 ± 1.01 | 96.79 ± 0.98 | 96.69 ± 1.01 |
| Arm perimeter (cm) | 29.50 ± 0.37 | 28.40 ± 0.38 | 28.44 ± 0.36 | 28.21 ± 0.36 |
| Leg perimeter (cm) | 36.64 ± 0.75 | 35.71 ± 0.59 | 35.49 ± 0.73 | 35.06 ± 0.57 |
| MNA (score) | 25.63 ± 0.49 | 24.72 ± 0.71* | 24.42 ± 0.39 | 25.50 ± 0.40*† |
| Values are expressed as mean ± standard error. Covariates: sex, age, frailty phenotype, BMI and educational level Abbreviations: SPPB; Short Physical Performance Battery; MVPA, Moderate to vigorous physical activity; LPA, Light physical activity; BMI, Body Mass Index; MNA, Mini Nutritional Assessment; * p<0.05 in relation to the pre-intervention moment in the same group; † p<0.05 in relation to control group in the same moment. | | | | |
