# Supplementary figures and images for: Effects of an educational intervention on frailty status, physical function, physical activity, sleep patterns, and nutritional status of older adults with frailty or pre-frailty: the FRAGSALUD study
Source: Front Public Health. 2023 Nov 30;11:1267666. doi: 10.3389/fpubh.2023.1267666 (PMC10720710; doi:10.3389/fpubh.2023.1267666)

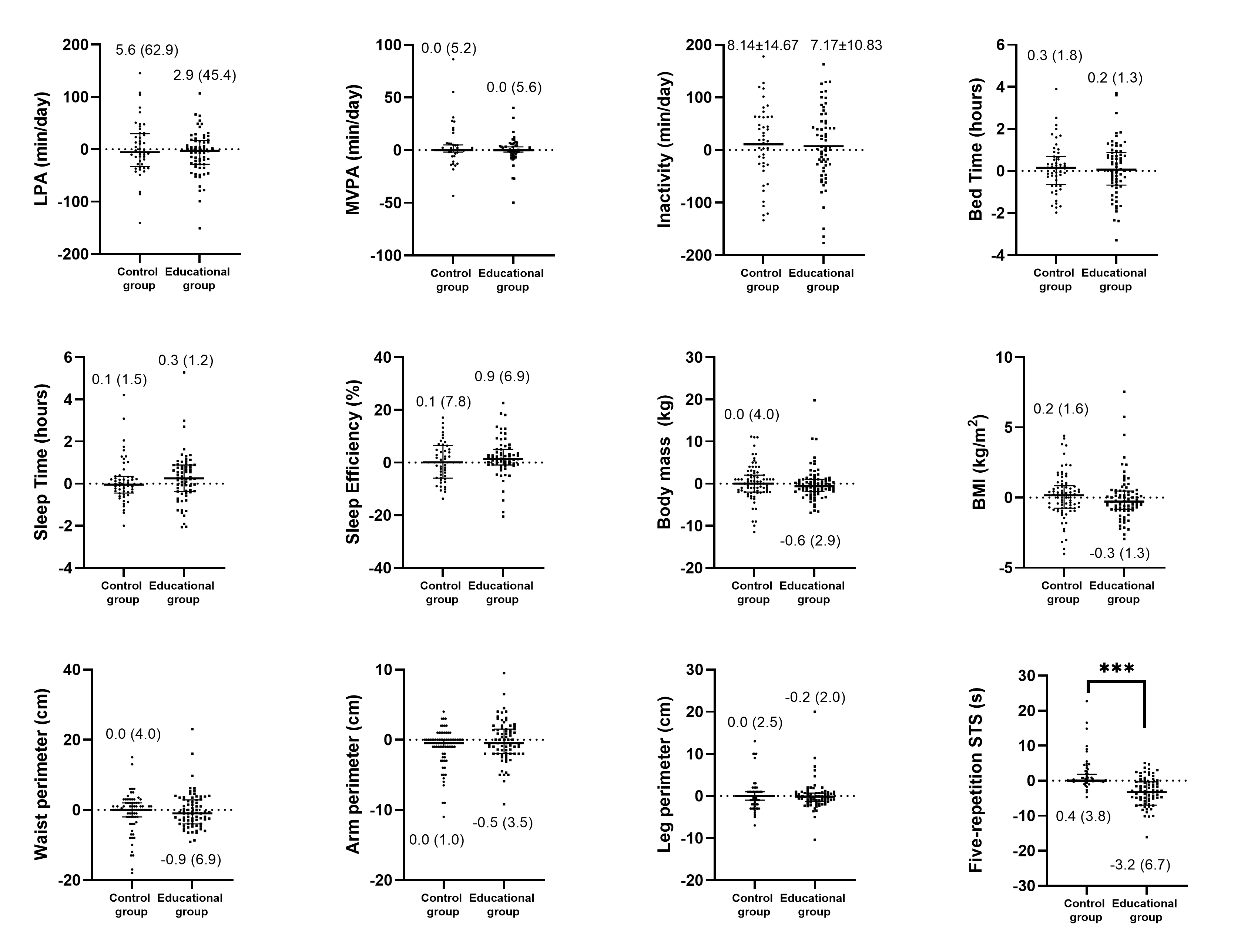

Supplement: Supplementary file 2 [file Image_1.TIF]

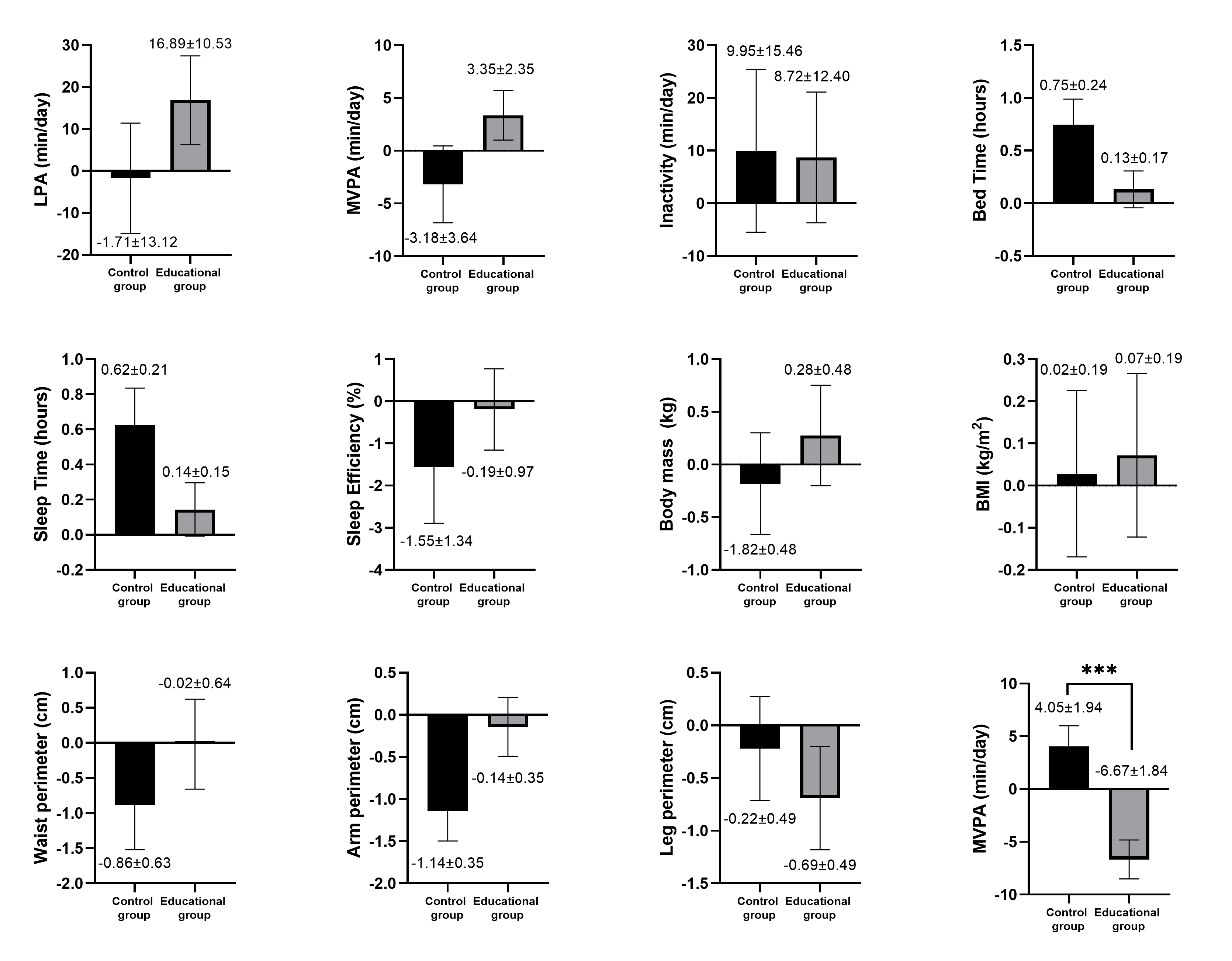

Supplement: Supplementary file 3 [file Image_2.TIF]
